# Quantification of left ventricular volumes and function in anesthetized beagles using real-time three-dimensional echocardiography: 4D-TomTec™ analysis versus 4D-AutLVQ™ analysis in comparison with cardiac magnetic resonance imaging

**DOI:** 10.1186/s12917-015-0568-5

**Published:** 2015-10-12

**Authors:** Judith Eskofier, Patrick Wefstaedt, Martin Beyerbach, Ingo Nolte, Stephan O. Hungerbühler

**Affiliations:** 3Small Animal Hospital, University of Veterinary Medicine Hannover, Foundation, Bünteweg, 30559 Hannover Germany; Department of Biometry, Epidemiology and Information Processing, University of Veterinary Medicine Hannover, Foundation, Bünteweg, 30559 Hannover Germany

## Abstract

**Backround:**

Real-time three-dimensional echocardiography (RT3DE) enables accurate volume determination of the left ventricle (LV), since measurements in foreshortened depicted views are avertable. Different analyzing programs are available for this RT3DE. The commonly used semi-automatic software 4D-AutLVQ™ showed underestimation of LV volumes in comparison with CMRI in healthy anesthetized dogs (Am J Vet Res 74(9):1223–1230, 2013). TomTec 4D LV-Function™ is an offline analysis program for morphological and functional analyses of the left ventricle by using manual measurement optimization, showing excellent agreement with CMRI in human medicine (Echocardiography 27(10):1263–1273, 2010; Eur J Echocardiogr 11(4):359–368, 2010; Echocardiography 24(9):967–974, 2007). The aim of the present study was to compare these different RT3DE analyzing software programs to test the possibility of one performing better than the other by assessing accuracy and reproducibility in comparison with the reference method cardiac magnetic resonance imaging (CMRI) by determining the left ventricular end-diastolic volume (EDV), end-systolic volume (ESV), stroke volume (SV) and ejection fraction (EF).

RT3DE and CMRI were performed during anesthesia in 10 healthy beagles. The analyzing programs 4D-AutLVQ™ (based on semi-automated border detection) and TomTec 4D LV-Function™ (primary manual tracking with semi-automated border detection) were used for RT3DE volume analysis of the left ventricle. Left ventricular EDV, ESV, SV and EF were measured and compared to those measured by the reference method CMRI. Repeated measurements were performed to determine inter- and intra-observer variability.

**Results:**

Both, 4D-AutLVQ™ and 4D-TomTec™ showed small but significant underestimation for EDV and ESV with quite good correlation (*r* = 0.34–0.69) in comparison with CMRI, without significant difference between each of them. Ejection fraction (EF) measured by 4D-TomTec™ showed no significant differences compared to CMRI (*p* = 0.12), while 4D-AutLVQ™ significantly underestimated LV-EF (*p* = 0.03). Analyzing time was shorter using 4D-AutLVQ™ compared to 4D-TomTec™. The inter-observer variability was higher using 4D-TomTec™ than with 4D-AutLVQ™, whereas both methods present excellent intra-observer variability.

**Conclusion:**

4D-TomTec™ and 4D-AutLVQ™ are feasible RT3DE analyzing programs, allowing accurate volume quantification of the left ventricle, albeit with significant underestimation of ventricular volumes in comparison with the gold standard CMRI. 4D-AutLVQ™ is performed faster with less inter-observer variability than 4D-TomTec™. Therefore, 4D-AutLVQ™ is the more practicable measurement method when comparing the different analyzing programs.

## Background

Acquired heart disease is common in veterinary medicine. Echocardiography is a commonly used technique in routine veterinary cardiology to diagnose congenital as well as acquired heart diseases [1]. One important feature to classify the severity of heart diseases in dogs is the echocardiographic volume estimation of the left ventricle (LV), as the left ventricle dilates through volume overload in the presence of progressive heart disease [2]. Conventionally used one-dimensional and two-dimensional echocardiography fails to provide the basis for accurate volume determination, caused by the accidental use of foreshortened views and moreover the dependence on elliptical geometric modeling of the anatomical LV shape [3–5]. The most accurate technique and proposed reference method for LV volume estimation is cardiac magnetic resonance imaging (CMRI) [6]. Recent advances in stronger gradients, faster imaging sequences and more homogeneous fields allow a three-dimensional visualization of the left ventricle in high spatial and temporal resolution as well as high image quality with excellent tissue contrast [7–9]. Furthermore, CMRI is a minimal invasive diagnostic imaging technique, allowing multiplanar imaging in any cardiac direction without limitations [10, 11]. However, high investment costs, the lack of veterinary expert knowledge and furthermore the need for general anesthesia limit the routine use in veterinary practice [8]. Nowadays, modern matrix-array transducer technology enables a dynamic, real-time three-dimensional reconstruction of the left ventricular by ultrasound, which is often denoted as 4D-echocardiography [12, 13]. Prior to this, human studies demonstrated accurate results with excellent correlations for volume estimation using real-time three-dimensional echocardiography (RT3DE) in comparison with CMRI in patients with cardiac disease [14, 15]. A study in healthy anesthetized dogs showed underestimation of LV volumes and less good correlations with RT3DE using 4D-AutLVQ™ in comparison to CMRI [3]. 4D-AutLVQ™ is a commercially available analysis software, working with semi-automatic tracking algorithms and simplified options for manual optimization of the LV cast. We hypothesize that improvements in measurement can be achieved by optimized border detection of the left ventricle using more precise software. TomTec 4D LV-Function™ is an offline analysis program for morphological and functional analyses of the left ventricle, which demands manual tracking of the endocardial border with add-on semi-automated border detection for generating 3D models of the LV cavity with intensive options for optimization of the LV volume cast. Previously published studies in human medicine confirmed excellent agreement in LV volume quantification with CMRI [16–18]. To the authors’ knowledge no study has been published in veterinary medicine comparing this method with the gold standard CMRI. Furthermore, to our knowledge, no study has been published comparing different offline RT3DE analysis software programs with CMRI in dogs. Therefore the aim of this study was to compare these two analyzing programs using the reference method CMRI, in order to evaluate if optimized contour detection could result in less underestimation of LV-volumes in healthy anesthetized beagles than shown previously by means of 4D-AutLVQ™ [3].

## Methods

### The animal model

This study was permitted by the Ethical Committee of the Lower Saxony State Office for Consumer Protection and Food Safety (33.9-42502-05-11A133). 10 healthy beagles, owned by the Small Animal Hospital of the University of Veterinary Medicine Hannover, were used in this study (3 females, 4 neutered males, 3 intact males, age: 6.8 ± 3.3 years, weight: 16.5 ± 1.8 kg). To ensure healthiness each dog underwent an extensive preliminary examination, including general examination, chest-radiography, electrocardiogram (ECG), blood pressure (BP) measurement, standard echocardiography and blood examination including CPC and serum chemical analyses.

On examination day, each dog underwent an echocardiographic examination following CMRI procedure. Each dog was premedicated with diazepam^1^ (0.1 mg/kg, intravenously) and levomethadone^2^ (0.08 mg/kg, intravenously). Propofol^3^ (up to 0.5 mg/kg, intravenously) was used for anesthetic induction. Subsequently, the dogs were orotracheally intubated using a cuffed tube^4^. Anesthesia was preserved with an isoflurane-oxygen-mixture^5^ with an end-tidal concentration of 1.5 %. During echocardiographic examination, the dog breathed spontaneously, but the data acquisition was performed in between breaths. For CMRI procedure, respiration was maintained mechanically using a respiratory system^6^ with a breathing frequency of 12 breaths per minute. Adjustment in respiratory volume was necessary to maintain an end-tidal PaCO_2_ of between 40 and 45 mmHg. Isoflurane concentration and oxygen saturation were continuously measured with a calibrated monitor.^7^ Heart rate was recorded by ECG. Each dog was given a continuous intravenous drip infusion^8^ (5 ml/kg/h), in order to guarantee equal hemodynamic conditions during anesthesia. The main examination time during anesthesia took 120 min. During the recovery phase the dogs were monitored continuously, including regular rectal temperature control and intravenous intake of fluids^h^ (5 ml/kg/h). The dogs were kept warm by underfloor heating and a heating jacket.^9^

### Echocardiography

Echocardiographic examinations were performed by one investigator (S.O.H.), using a commercially available ultrasound unit,^10^ equipped with a matrix-array 3 V transducer for three-dimensional echocardiography (3DE) and ECG-monitoring. The dogs were positioned in left lateral recumbency on a raised table with central opening specially designed for veterinary echocardiographic examinations. The recorded images and loops were digitally stored in DICOM-format and sent to a separate workstation, equipped with two different commercially available software programs^11^,^12^ for offline analysis. To assess inter-observer variability, end-diastolic volume (EDV), end-systolic volume (ESV) and ejection fraction (EF) were measured by two independent investigators (J.E. and S.H.) with different levels of experience on separate days using both analyzing software programs. Four weeks later, measurements were repeated in random order by one investigator (J.E), in order to evaluate intra-observer variability. To assess duration of the analysis, a stopwatch was started with the first manual intervention of the selected three-dimensional data-set and stopped with completion of the volume analysis. Each measurement was repeated three times and averaged for further statistical analysis.

### RT3DE Image Acquisition

**Fig. 1:**
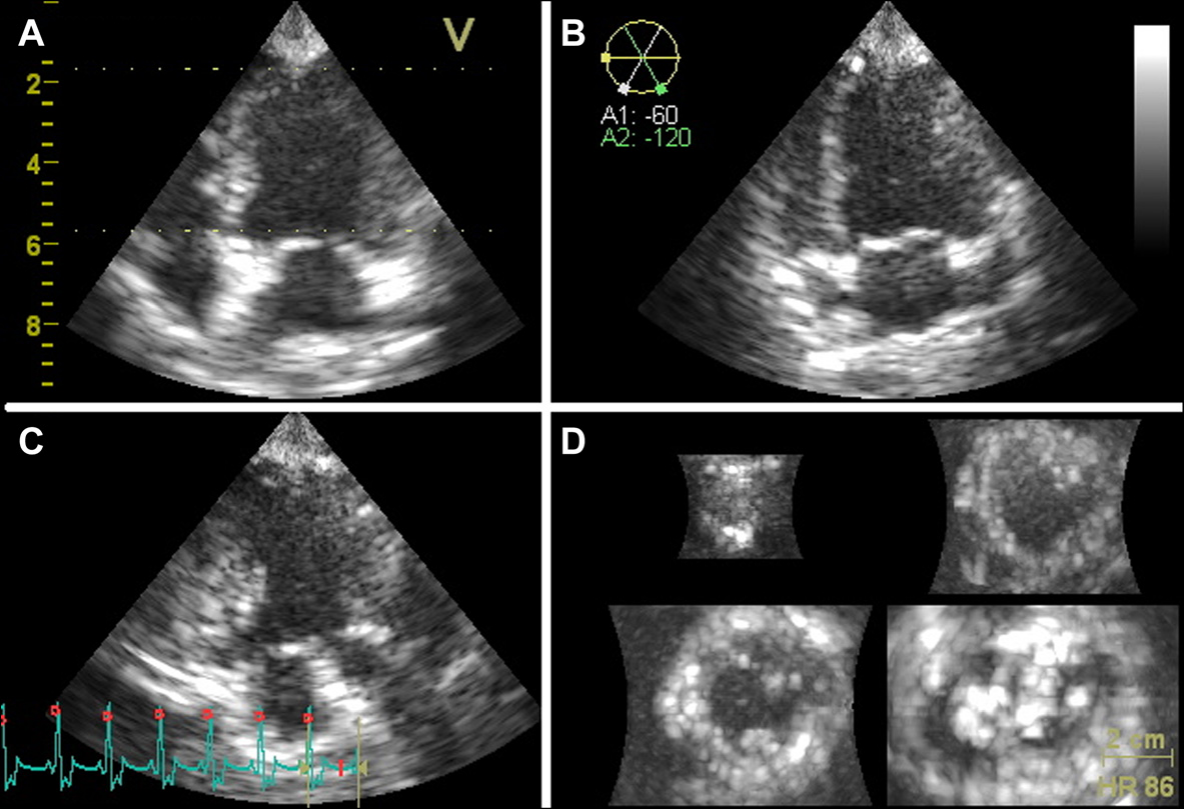
Representative RT3DE image of the left ventricle of a healthy beagle. Using RT3DE-mode the left ventricle is simultaneously displayed in three different apical planes and a short axis plane. To avoid foreshortening, automatic and manual adjustment was necessary. **a** left apical 4 chamber view, **b** left apical 2 chamber view and **c** left apical 3 chamber view, **d** short-axis views of different levels of the left ventricle

RT3DE image acquisition was performed as recommended in the Guidelines of the American Society of Echocardiography from left apical using harmonic mode [19]. Sound frequency, image contrast, penetration depth and image size were adjusted to obtain optimal visualization of the left ventricle. To include the entire LV cavity within the pyramidal RT3DE dataset, the left ventricle was recorded over several heart beats in four wedge-shaped sub-volumes, which were subsequently merged by R-wave triggering. Penetration depth and angle scan sector were adjusted to a minimum, still encompassing the entire left ventricle. The frame rate of RT3DE data-sets reached from 30 to 50 beats per second. In the following, the left ventricle was displayed in a quad-screen in different apical and short axis views (Fig. 1). Both analyzing software programs were given the same RT3DE data-set.

### 4D-TomTec™ analysis

**Fig. 2:**
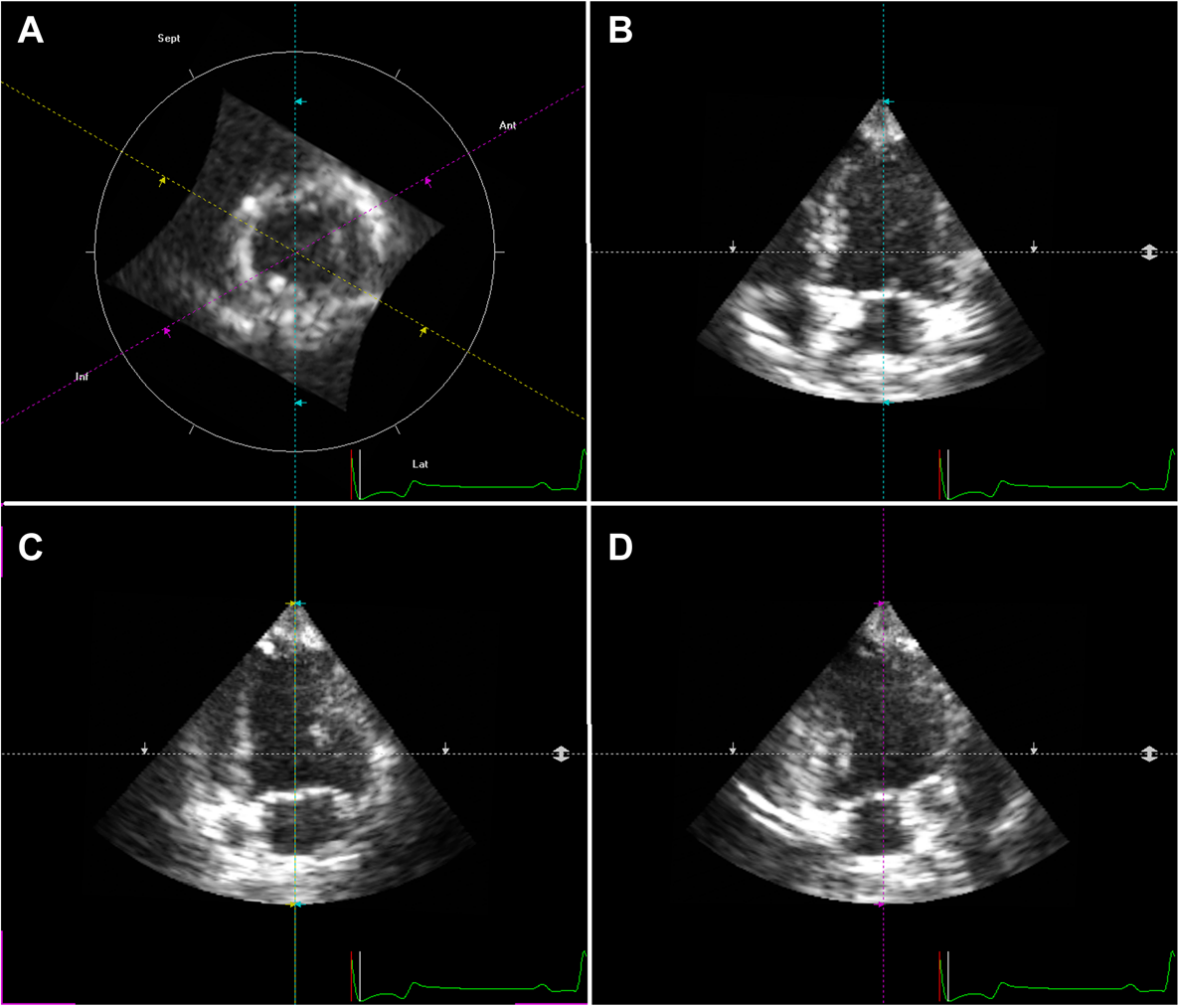
Representative illustration of the left ventricle of a healthy beagle starting the analyzing program 4D-TomTec™. The left ventricle is simultaneously displayed in a quad-screen in different planes. To avoid foreshortening, manual adjustment was necessary. **a** dynamic short-axis view, **b** left apical 4 chamber view, **c** left apical 2 chamber view, and **d** left apical 3 chamber view

**Fig. 3:**
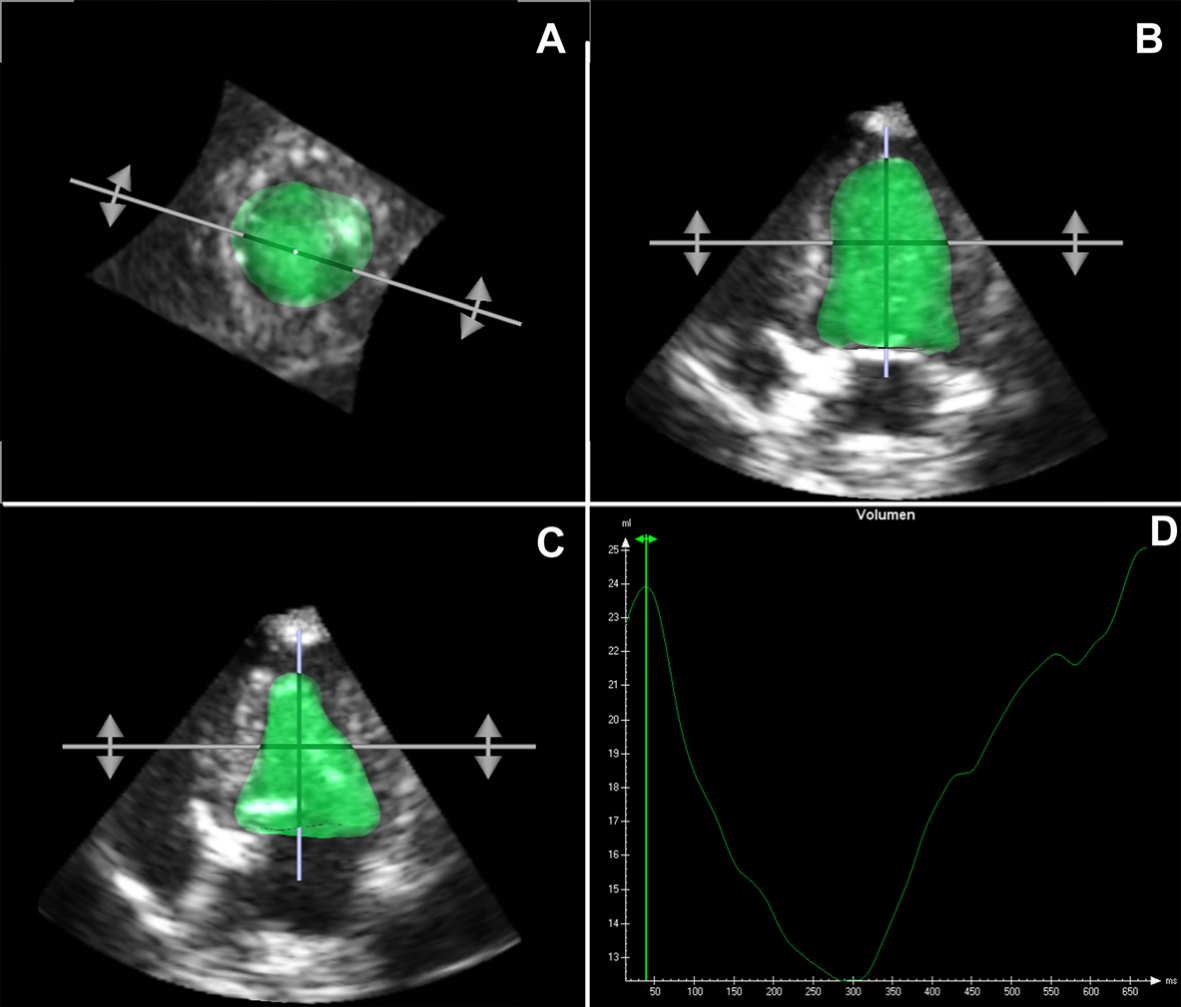
Representative RT3DE analysis of the left ventricle of a healthy beagle using 4D-TomTec™ software. The image shows a dynamic geometric model of the left ventricle cavity in different views as well as a volume-time curve. **a** dynamic short-axis view, **b** end-diastolic volume cast, **c** end-systolic volume cast and **d** volume-time curve

RT3DE volume analysis was performed offline using TomTec 4D LV-Function™ software^l^. The software was purchased in addition to the software of the ultrasound device and was integrated into the workstation (EchoPAC). Therefore measurements could be performed with the same data sets as used for 4D-AutLVQ™. Starting the analyzing program, the left ventricle was displayed in four different views in a quad-screen (Fig. 2). In order to obtain the maximal LV long-axis view and to avoid foreshortening, manual adjustment was necessary in the left apical 4-chamber (4Ch), 2-chamber (2Ch) as well as 3-chamber (3Ch) view. Adjustment in one window automatically changed views in the other windows of the quad-screen. Subsequently, end-systole and end-diastole were defined automatically and displayed in the quad-screen. Manual contouring of the endocardial borders in all three long-axis planes in end-systole and end-diastole was performed. Papillary muscles and trabeculae were included in the LV volume in all performed measurement methods. Starting the automatic contour detection the program created a three-dimensional volume cast and a volume curve by tracking the endocardial border of the whole heart and through the entire cardiac cycle aligned by the manual contouring. In the following manual volume cast correction was performed frame by frame for optimal adaption to the endocardium. After perfect adjustment to the endocardial border in all planes and all phases of the cardiac cycle, the volume curve was finally evaluated (Fig. 3). Here, the smallest volume was defined as ESV and the highest volume as EDV. EF was calculated as follows: ((EDV-ESV)/EDV)*100 and SV as: EDV-ESV = SV. Three consecutive heart cycles were measured and the results averaged.

### 4D-AutLVQ™ analysis

**Fig. 4:**
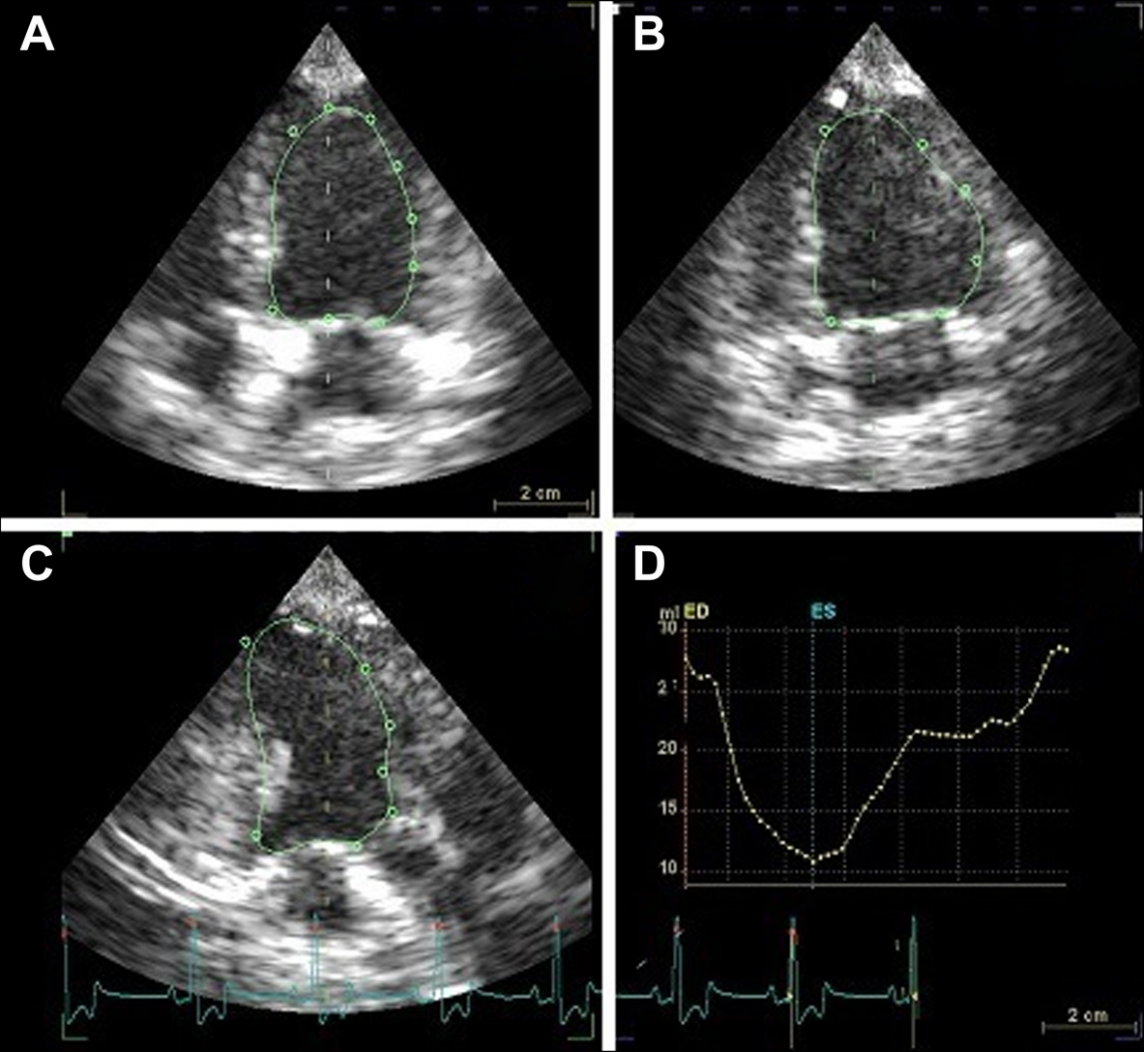
Representative RT3DE analysis of the left ventricle of a healthy beagle using 4D-AutLVQ™ software. The image shows a dynamic geometric model of the left ventricle cavity in different views. **a** left apical 4 chamber view, **b** left apical 2 chamber view, **c** left apical 3 chamber view, **d** volume-time curve

Furthermore, 4D-AutLVQ™ software^13^ was used for RT3DE volume determination of the left ventricle. First, manual adjustment in all three apical views was necessary to obtain the maximal LV long-axis and to avoid foreshortening. End-diastole and end-systole were automatically identified and manually adjusted if necessary for optimal depiction of the left ventricle. End-diastole was defined as first picture with closed mitral valve and end-systole as the frame directly before the mitral valve opened again as proposed by the American Society of Echocardiography [20]. Placing an identification point at the middle of the LV base and a second point at the LV apex in one of the apical LV views in end-diastole and end-systole, automatically started the endocardial border detection. Manual correction was necessary in all cases in order to place the boundary as close as possible near the endocardium and to include papillary muscles and trabeculae in the LV volume calculation. Setting of new marker points in a specific heart phase altered the volume cast of the whole heart automatically. A whole endocardial border tracing was not possible. The optimization required multiple interventions to achieve the best fit. After final adjustment the detection of the volume curve was started. Now the automatically generated volume cast was shown as a yellow line in all phases of the heart cycle as well as a volume time curve (Fig. 4). In the following, the program automatically constructed a dynamic model of the LV cavity and computed EDV and ESV. The smallest left ventricular volume was defined as ESV and the highest volume as EDV. EF and SV were calculated as mentioned above. Three consecutive heart cycles were measured and the results averaged. Furthermore, an analysis of the same data-set was performed without manual correction of the LV volume. Therefore the marker points were positioned as above, but no further adaption was performed. The volume curve was evaluated as described above.

### CMRI preparation

All dogs were shaved at the left side of the thorax to adhere special MRI-compatible electrocardiographic electrode pads^14^ on the dog’s chest, in a right-angled array. For further CMRI-procedure, the dogs were positioned in a headward supine position. Overlapping surface coil-units^15^ were applied around the thorax of the dogs at the level of the heart. All dogs were equipped with earplugs to reduce the noise of the gradients during CMRI acquisition and were kept warm with heated gel cushions.

### CMRI imaging and analysis

**Fig. 5:**
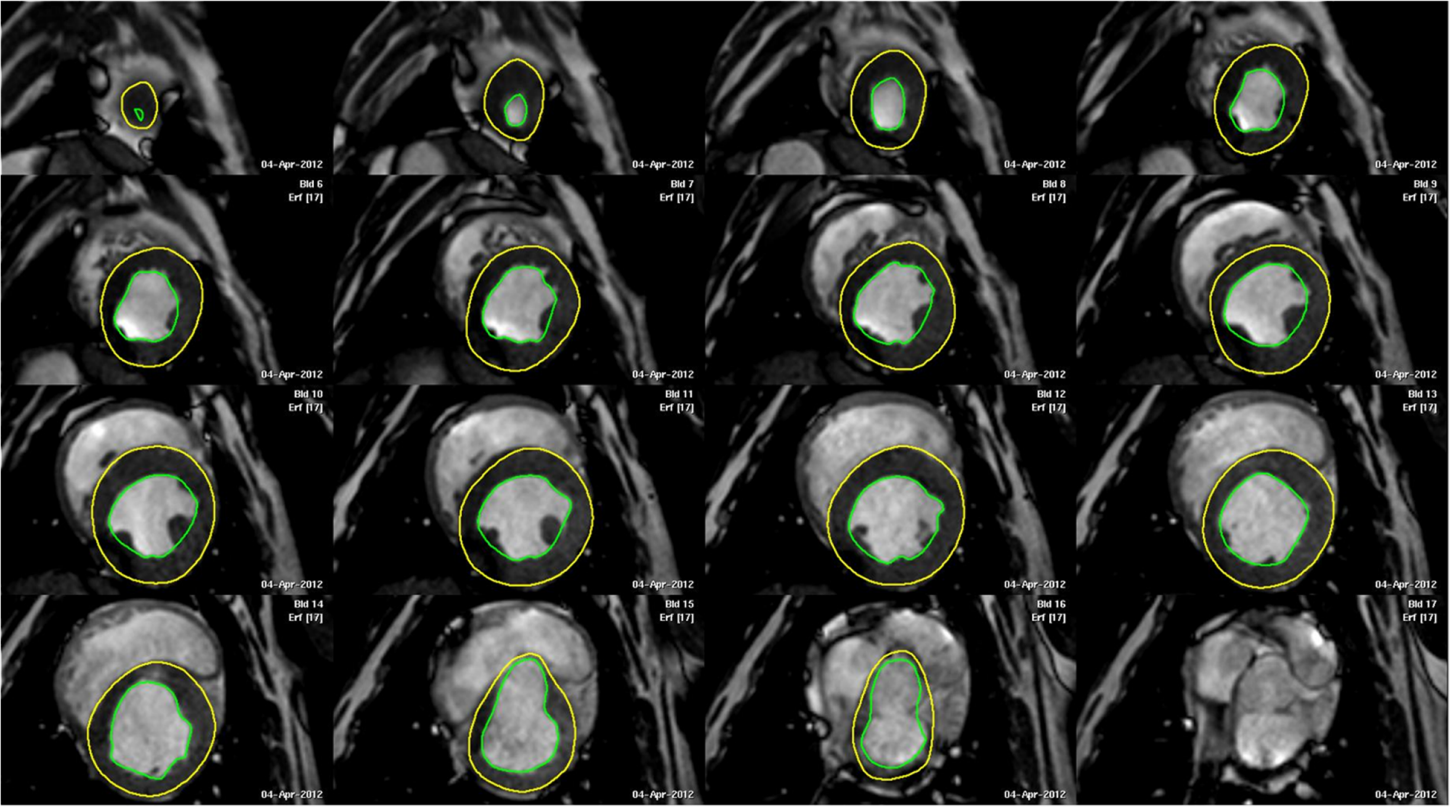
Representative CMRI analysis of the end-diastolic volume of the left ventricle of a healthy beagle. Manual contouring of the LV epi- and endocardial borders was done in each short-axis slice in end-diastole as well as in end-systole. Papillary muscles were included in the LV volume. Slices at the level of the heart base were included if they were surrounded by 50 % or more of ventricular myocardium

CMRI was performed using a modern 3 T scanner.^16^ To reduce breathing-artifacts, manual respiration was turned off during acquisition (maximal 30 s). Before starting each scanning sequence, heart rate was updated on the ECG gating system. Retrospective ECG-triggering was used in order to reduce cardiac motion artifacts. CMRI was performed as previously described [3]. A three-plane localizer was used to plan CMRI acquisition. A steady state free precession sequence (FFE) was applied to create 22 contiguous short-axis images of the heart perpendicular to the longitudinal axis of the left ventricle (4 mm slice-thickness respectively; without any gap). Acquisition parameters were adjusted as follows: field of view = 320 × 198 × 88 mm, repetition time = 4 ms, echo time = 2 ms and pulse flip angle = 40°). All images and cine-loops (30 frames per cardiac cycle) were digitally stored and sent to a commercially available workstation,^17^ using a special software program^18^ for cardiac analyses. All CMRI measurements were performed by the same observer (J. E.). End-diastole was defined as the largest LV cavity, while end-systole was defined as the smallest LV cavity. Starting the CMRI analyzing program, end-diastole and end-systole were automatically detected according to the ECG and were confirmed by visual validation of the largest respectively smallest LV cavity. Manual contouring of the LV endocardium and epicardium was performed in both, end-diastole and end-systole, for each slice from the LV apex to the mitral annulus (Fig. 5). Papillary muscles and trabeculae were included in the LV volume calculation. Slices were included in the LV volume calculation if the ventricle was surrounded by more than 50 % of myocardium (end-diastole: 15 ± 1 slices, end-systole: 13 ± 1 slices). EDV and ESV were geometrically independently calculated by the software program, multiplying the area within the endocardial tracing with the respective slice thickness and subsequent volume summarizing all short-axis slices [7]. EF and SV were calculated as described above.

### Statistical analysis

Commercially available software programs^19^,^20^ were used for statistical analysis. EDV, ESV, and EF values were expressed as mean ± standard deviation (SD). To verify normal distribution of data, a Shapiro-Wilk test was performed. In the following, a single factor variance analysis was used to test the null hypothesis that both RT3DE analyzing software programs provide identical results measuring LV volumes and function as compared to CMRI. Multiple pairwise comparisons between the analyzing methods were performed to examine whether significant differences occur using a paired *t*-test for the normally distributed data. A P-value of < 0.05 was considered significant. For each pair of values, limits of agreement and systematic errors were assessed by evaluating the mean difference (bias) and the standard deviation of the differences using the Bland-Altman method. The strength of relation between each RT3DE analyzing technique and CMRI reference values was expressed by Pearson correlation. Correlations were defined as excellent with r ≥ 0.9, very good with *r* < 0.9 and ≥ 0.7, good with *r* < 0.7 and ≥ 0.5, less good *r* < 0.5 and ≥ 0.3 and weak with *r* < 0.3. Inter-observer and intra-observer variability was expressed as mean difference ± SD. An intra-class correlation coefficient was calculated and assessed using the following scheme: ≤ 0.1 poor agreement, > 0.1 and ≤ 0.2 weak agreement, > 0.2 and ≤ 0.4 moderate agreement, > 0.4 and ≤ 0.6 average agreement, > 0.6 and ≤ 0.8 good agreement, > 0.8–1.0 almost perfect agreement. Values of time exposure for RT3DE analyses were assessed using a paired *t*-test and were expressed as mean ± SD.

## Results

**Fig. 6:**
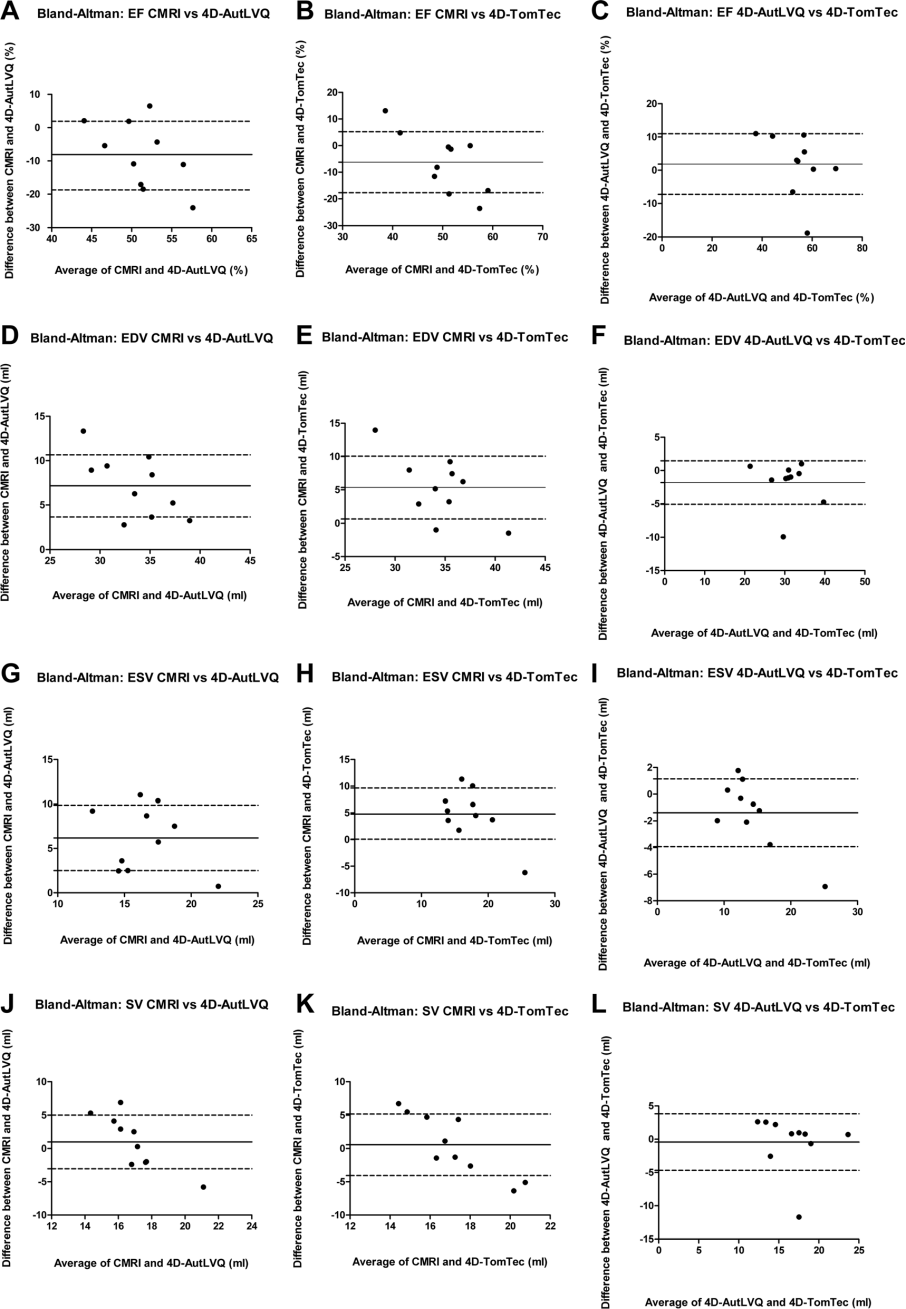
Bland-Altman analysis left ventricular volumetric measurements. Bland-Altman analysis plots of the differences between end-diastolic volume (EDV), end-systolic volume (ESV), stroke volume (SV) and ejection fraction (EF) determined by the use of cardiac magnetic resonance imaging (CMRI) with 4D-AutLVQ™ (AutLVQ) and 4D-TomTec™ (TomTec), two different real-time three-dimensional echocardiography analyzing softwares, of 10 healthy beagles. The solid and dotted lines represent mean value of differences and limits of agreement (bias ± 1.96 standard deviation), respectively. **a**: EF CMRI vs AutLVQ, **b**: EF CMRI vs TomTec; **c** EF TomTec vs AutoLVQ; **d** EDV MRT vs AUTLVQ; **e** EDV CMRI vs TomTec; **f** EDV AutoLVQ vs TomTec; **g** ESV CMRI vs AutLVQ; **h** ESV CMRI vs TomTec; **i** ESV AUTLVQ vs TomTec; **j** SV CMRI vs AutLVQ; **k** SV CMRI vs TomTec; **l** SV AutLVQ vs TomTec

The blood parameters of all examined dogs were in the normal range. X-rays showed no signs of congestion and the heart size was normal [21] with a mean vertebral heart score of 10.5 (range: 10.0 to 11.2). Furthermore, the BP measurement with mean systolic BP of 153.3 ± 13.8 mmHg and mean diastolic BP of 88.1 ± 13.5 mmHg was in the normal range as well [22]. The heart rate was not significantly different between the methods (echocardiographic examination: 87.9 ± 9 beats per minute (bpm), CMRI 89.8 ± 9.11 bpm). Data acquisition and volume analysis were successful in all ten healthy anesthetized beagles with values presented in Table 1. The results of statistical analysis are shown in Table 2 as well as in Fig. 6.

**Table 1:** Values of left ventricular volumetric measurements

	CMRI	4D-AutLVQ™	4D-TomTec™	4D-AutLVQ™
		with manual correction		without manual correction
EF (%)	47.22 ± 4.45^a,d^	55.30 ± 7.96^a^	53.44 ± 11.40^b,f^	56.70 ± 6.79^d,f^
EDV (ml)	37.14 ± 2.69^a,b,d^	29.97 ± 4.75^a,e^	31.78 ± 5.35^b,f^	25.23 ± 7.33^d,e,f^
ESV (ml)	19.68 ± 2.82^a,b,d^	13.50 ± 3.53^a,e^	14.89 ± 5.52^b,f^	11.20 ± 4.14^d,e,f^
SV (ml)	17.46 ± 1.10^d^	16.47 ± 3.63^e^	16.89 ± 4.24^f^	14.03 ± 3.81^d,e,f^

Mean and standard deviation (SD) of left ventricular end-diastolic (EDV) end-systolic (ESV) volume, stroke volume (SV) and ejection fraction (EF) in 10 healthy anesthetized beagles measured with different three-dimensional echocardiographic based analyzing software programs (4D-AutLVQ™ and 4D-TomTec™) in comparison with cardiac magnetic resonance imaging (CMRI)

^a,b,c^significant differences (*P-*value < 0.05); ^a^significant differences between CMRI and 4D-AutLVQ™ with manual correction; ^b^significant differences between CMRI and 4D-TomTec E; ^c^significant differences between 4D-AutLVQ™ and 4D-TomTec™, ^d^significant differences between CMRI and 4D-AutLVQ™ without manual correction; ^e^significant differences between 4D-AutLVQ™ with manual correction and 4D-AutLVQ™ without manual correction; ^f^significant differences between 4D-TomTec™ and 4D-AutLVQ™ without manual correction

**Table 2:** Overview of statistical analysis

	EF					EDV					EDV					ESV				
	Correlation		Bland Altman		GC	Correlation		Bland Altman		GC	Correlation		Bland Altman		GC	Correlation		Bland Altman		GC
	r	*P*- value	Bias	SD	*P*-value	r	*P*- value	Bias	SD	*P*-value	r	*P* value	Bias	SD	*P*-value	R	*P* value	Bias	SD	*P*-value
Methods compared																				
CMRI vs 4D-TomTec™	−0.24	0.5117	−8.08	9.99	0.1199	0.69	**0.0273**	7.17	3.49	**0.0057**	0.34	0.3311	6.18	3.68	**0.0124**	−0.24	0.5013	0.99	4.03	0.7072
CMRI vs 4D-AutLVQ™	0.18	0.1613	−6.22	11.45	**0.0308**	0.48	0.1644	5.36	4.70	**0.0001**	0.48	0.1644	4.79	4.86	**0.0005**	−0.24	0.5127	0.57	4.63	0.4570
4D-TomTec™ vs	0.61	0.0625	1.86	9.12	0.5355	0.80	**0.0056**	−1.81	3.26	0.1120	0.94	**<0.0001**	−1.39	2.54	0.1178	0.42	0.2220	−0.42	4.25	0.7598
4D-AutLVQ™																				

Results of statistical comparisons between the different three-dimensional echocardiographic based volumetric analyzing software programs (4D-TomTec™, 4D-AutoLVQ™) and cardiac magnetic resonance imaging (CMRI) used to calculate left ventricular end-diastolic (EDV) end-systolic (ESV) volume, stroke volume (SV) and ejection fraction (EF) in 10 healthy anesthetized beagles (*GC* Group comparison with paired *T*-Test, *SD* Standard Deviation, *r* Pearson regression coefficient). Bold letters illustrate significant differences (*P* < 0.05)

### EDV

In comparison with CMRI (EDV = 37.14 ± 2.69 ml) there was significant underestimation of EDV with 4D-TomTec™ (*p* = 0.0057, bias 7.17) and 4D-AutLVQ™ (*p* = 0.0001, bias 5.36). Correlation with CMRI was stronger with 4D-TomTec™ (*r* = 0.69) than with 4D-AutLVQ™ (*r* = 0.48). The underestimation was slightly but not significantly less with 4D-TomTec™ (EDV = 31.78 ± 5.35) than with 4D-AutLVQ™ (EDV = 29.97 ± 4.75) with small bias (*p* = 0.1120, bias −1.81) and very good correlation (*r* = 0.80) when comparing the echocardiographic methods.

### ESV

In comparison with CMRI (ESV = 19.68 ± 2.82 ml) there was significant underestimation of ESV with 4D-TomTec™ (*P* = 0.0124, bias 6.18) and 4D-AutLVQ™ (*p* = 0.0005, bias 4.79). In contrast to the measurement of EDV the correlation was stronger with 4D-AutLVQ™ (*r* = 0.48) than with 4D-TomTec™ (*r* = 0.34). The underestimation was slightly but not significantly less with 4D-TomTec™ (ESV = 14.89 ± 5.52) than with 4D-AutLVQ™ (ESV = 13.50 ± 3.53) with small bias (*p* = 0.1178, bias −1.39) and excellent correlation (*r* = 0.94) when comparing the echocardiographic methods.

### EF

In comparison with CMRI (EF = 47.22 ± 4.45) 4D-AutLVQ™ measurements showed significantly higher EF values (EF = 55.30 ± 7.96, *p* = 0.0308, bias −6.22) and weak correlation (*r* = 0.18). 4D-TomTec™ values (EF = 53.44 ± 11.40, *p* = 0.1199, bias −8.08) were not significant higher than CMRI with weak correlation (*r* = −0.24) as well. There was no difference and small bias (*p* = 0.5355, bias 1.86) and good correlation (*r* = 0.61) when comparing the echocardiographic methods.

### SV

In comparison with CMRI (SV = 17.46 ± 1.10 ml) there was no significant difference for SV either with 4D-TomTec™ (*p* = 0.7020, bias 0.99) or with 4D-AutLVQ™ (*p* = 0.4570, bias 0.57). CMRI measurements correlated weakly with 4D-AutLVQ™ (*r* = −0.24) and with 4D-TomTec™ (*r* = −0.24). Furthermore, there was no difference when comparing both echocardiographic methods (*p* = 0.7598, bias – 0.42) with less good correlation (*r* = 0.42).

### Time for volume analysis

The time needed for volume-analysis (Table 3) was significantly (*p* < 0.0001) shorter using 4D-AutLVQ™ (145 ± 57 sec) compared to 4D-TomTec™ (215 ± 46 sec). If 4D-AutLVQ™ was carried out without manual correction, the measurement time was reduced even more (48.54 ± 23.10, *p* < 0.0001) to approximately a fourth of the time using 4D-TomTec™.

**Table 3:** Comparison of measurement duration

Method	Mean	SD	Min	Max
4D-TomTec™	214.94	46.06	118.00	306.00
4D-AutLVQ™ with manual correction	145.21	57.03	72.00	365.00
4D-AutLVQ™ without manual correction	48.54	23.10	24.00	125.00

Mean and standard deviation (SD) as well as minimum (Min) and maximum (Max) of analyzing time needed to perform a volumetric measurement in 10 healthy anesthetized beagles needed by two different three-dimensional echocardiography based volumetric analyzing software programs (sec = seconds). Furthermore analysis time of 4D-AutLVQ™ without manual correction. There is a significant (*P* < 0.0001) difference between each method

### 4D-AutLVQ™ without manual correction

Indeed, the volume quantification of 4D-AutLVQ™ without manual correction was significantly faster than with all other methods, but the left ventricular volumes (EDV, ESV and SV) were not only significantly lower compared to CMRI but also significantly lower than using the other echocardiographic methods (Tables 1 and 4). EF was highest compared with the other methods and significantly different from CMRI and 4D-TomTec, but not from 4D-AutLVQ™ regarding manual correction. The correlations with CMRI were comparable for 4D-AutLVQ™ with as well as without manual optimization.

**Table 4:** Statistical analysis of 4D-AutoLVQ™ without manual correction

	EF					EDV					ESV					SV				
	Correlation		Bland Altman		GC	Correlation		Bland Altman		GC	Correlation		Bland Altman		GC	Correlation		Bland Altman		GC
	r	*P* value	Bias	SD	*P*-value	r	*P*-value	Bias	SD	*P*-value	r	*P*-value	Bias	SD	*P*-value	r	*P*-value	Bias	SD	*P*-value
Methods compared																				
CMRI vs	0,15	0,6831	−9,48	7,55	**0,0032**	0,58	0,3346	11,91	6,18	**0,0002**	0,48	0,2268	8,48	3,74	**<0,0001**	0,08	**0,0057**	3,43	3,88	**0,0210**
4D-AutLVQ™ without manual correction																				
4D-AutLVQ™ with manual correction vs	0,46	0,2070	−2,70	6,05	0,1915	0,74	0,5435	7,17	5,11	**0,0016**	0,70	0.4923	3,73	2,99	**0,0033**	0,64	0,4149	3,44	2,94	**0,0049**
4D-AutLVQ™ without manual correction																				
4D-TomTec™ vs	0,28	0,4335	−7,02	6,96	**0,0110**	0,63	0,4028	10,52	5,76	**0,0003**	0,69	0,4815	6,84	2,98	**<0,0001**	0,19	**0,0351**	3,68	3,92	**0,0049**
4D-AutLVQ™ without manual correction																				

Results of statistical comparisons between cardiac magnetic resonance imaging (CMRI), 4D-TomTec™, 4D-AutoLVQ™ with manual correction and 4D-AutoLVQ™ without manual optimization of the values left ventricular end-diastolic (EDV) end-systolic (ESV) volume, stroke volume (SV) and ejection fraction (EF) in 10 healthy anesthetized beagles (*GC* Group comparison with paired *T*-Test, *SD* Standard Deviation, *r* Pearson regression coefficient). Bold letters illustrate significant differences (*P* < 0.05)

### Reproducibility

Regarding the inter-observer variability (Table 5) there were significantly different left ventricular volumes (EDV and ESV) between both observers using 4D-TomTec™, whereas 4D-AutLVQ™ showed only significant differences for EDV. The relative difference was greater with 4D-TomTec™ (EDV: 11.78 %, ESV: 19.14 %) than with 4D-AutLVQ™ (EDV: 7.80 %, ESV: 10.80 %). The EF and SV were comparable with an inter-observer variability < 8 %. The intra-class correlation coefficient was lower using 4D-TomTec™ (0.208–0.453) than using 4D-AutLVQ™ (0.243–0.657). 4D-TomTec™ as well as 4D-AutLVQ™ showed excellent intra-observer variability < 3.6 % and an almost perfect intra-class correlation coefficient (ICC > 0.8) with both observers (Tables 6 and 7).

**Table 5:** Inter-observer variability

Variable	First	Second	Relative Difference (%)	*P*-Values	Bias	ICC
	Observer	Observer				
4D-Auto-LVQ						
EDV (ml)	32.4 ± 4.2	30.0 ± 4.8	7.80	**0.0410**	2.43	0.657
ESV (ml)	14.9 ± 2.4	13.5 ± 3.5	10.80	0.1274	1.43	0.562
SV (ml)	17.5 ± 2.1	16.5 ± 3.6	5.89	0.3485	0.98	0.414
EF (%)	54.0 ± 2.8	55.3 ± 7.9	2.40	0.5927	−1.31	0.243
TomTec						
EDV (ml)	35.8 ± 3.6	31.8 ± 5.4	11.78	**0.0126**	3.98	0.453
ESV (ml)	18.0 ± 2.8	14.9 ± 5.52	19.14	**0.0380**	3.16	0.465
SV (ml)	17.7 ± 1.9	16.9 ± 4.2	4.77	0.5463	0.83	0.208
EF (%)	49.7 ± 4.33	53.4 ± 11.4	7.29	0.2627	−3.75	0.326

Results of statistical comparisons between repeated measurements by two observers of the different three-dimensional echocardiographic based volumetric analyzing software programs (4D-TomTec™, 4D-AutoLVQ™) used to calculate left ventricular end-diastolic (EDV), end-systolic volume (ESV), stroke volume (SV) and ejection fraction (EF) in 10 healthy anesthetized beagles. Bold letters illustrate significant differences (*P* < 0.05)

**Table 6:** Intra-observer variability observer one

Variable	First Observer	First Observer	Relative Difference (%)	*P*-Values	Bias	ICC
	First Measurement	Second Measurement				
4D-Auto-LVQ						
EDV (ml)	30.0	30.1	−0.6	0.8071	0.16	0.956
ESV (ml)	13.5	13.8	−2.4	0.4085	0.34	0.968
SV (ml)	16.5	16.3	1.0	0.7933	0.19	0.907
EF (%)	55.3	54.2	2.0	0.3992	1.11	0.936
TomTec						
EDV (ml)	31.8	31.7	0.1	0.9600	0.03	0.967
ESV (ml)	14.9	14.7	1.2	0.9698	0.15	0.954
SV (ml)	16.9	17.0	−0.8	0.8566	−0.15	0.892
EF (%)	53.4	53.7	−0.5	0.9027	−0.27	0.833

Results of statistical comparisons between repeated measurements by observer one at an interval of more than 2 weeks of the different three-dimensional echocardiographic based volumetric analyzing software programs (4D-TomTec™, 4D-AutoLVQ™) used to calculate left ventricular end-diastolic (EDV), end-systolic volume (ESV), stroke volume (SV) and ejection fraction (EF) in 10 healthy anesthetized beagles

**Table 7:** Intra-observer variability observer two

Variable	Second Observer	Second Observer	Relative Difference (%)	*P*-Values	Bias	ICC
	First Measurement	Second Measurement				
4D-Auto-LVQ						
EDV (ml)	32.4	32.7	−1.0	0.5558	−0.3	0.942
ESV (ml)	14.9	14.6	2.0	0.4932	0.3	0.935
SV (ml)	14.5	18.1	−3.6	0.1945	0.3	0.807
EF (%)	54.0	54.8	−1.5	0.4761	−0.6	0.823
TomTec						
EDV (ml)	35.8	35.5	0.7	0.6249	0.3	0.946
ESV (ml)	18.0	17.6	2.5	0.3117	0.4	0.949
SV (ml)	17.7	17.9	−1.0	0.7570	−0.2	0.789
EF (%)	49.7	50.5	−1.6	0.3527	−0.8	0.840

Results of statistical comparisons between repeated measurements by observer two at an interval of more than 2 weeks of the different three-dimensional echocardiographic based volumetric analyzing software programs (4D-TomTec™, 4D-AutoLVQ™) used to calculate left ventricular end-diastolic (EDV), end-systolic volume (ESV), stroke volume (SV) and ejection fraction (EF) in 10 healthy anesthetized beagles. Bold letters illustrate significant differences (*P* < 0.05)

## Discussion

RT3DE is a modern technique allowing volume determination of the left ventricle [3, 16]. Underestimation of LV volumes in comparison to CMRI in anesthetized healthy beagles was described recently using 4D-AutLVQ™ [3]. However different analyzing programs are available for RT3DE volume estimation which might be able to deliver more accurate results. In this context 4D-TomTec™ could be a more suitable program as it uses a different contour detection algorithm with more detailed options for manual optimization of endocardial border demarcation than 4D-AutLVQ™. Thus the purpose of this study was to compare these two analyzing programs with the reference method CMRI, to evaluate whether optimized contour detection can result in less underestimation of LV-volumes in healthy anesthetized beagles.

### LV volumes

In this study, both analyzing programs show small but significant underestimations when measuring EDV and ESV, most likely caused by worse spatial and temporal resolution than the reference method CMRI [3, 23]. Reduced temporal resolution can lead to missing of correct maximal or minimal volume. Using sub-volume generated data-sets of the LV in this study enabled records of the LV with a temporal resolution between 30 and 50 frames/s as advised for RT3D volumetric measurements in human medicine [24, 25]. This high frame rate was generated by using 4 sub-volumes of 4 consecutive heart cycles. The achieved frame rate of approximately 60 frames per heart cycle is quite high, considering that CMRI generates 30 phases per heart cycle. In RT3DE data acquisition care was taken, that the beat-to-beat rate did not differ between these heart cycles, but small alterations could not be avoided which leads to stitching artifacts of the three-dimensional data-set. Furthermore, movement of the thorax can lead to less good spatial resolution. This was not a problem in the examined anesthetized dogs, but could prove to be a problem in awake dogs. Moreover, it cannot be completely ruled out, that the negative inotropic effect of the prolonged isofluran anesthesia [26] could result in higher LV volumes measured by CMRI, since RT3DE was performed first. Also, a differing patient positioning could influence the results of this study. A lower stroke volume caused by reduced ventricular filling is described by catheter intervention or thermodilution technique [27–29] in anesthetized animals in supine position. However, in this present study the lowest LV volumes were measured by RT3DE methods in lateral recumbency. Therefore, a possible positioning associated effect is probably concealed by the methodical difference. In conclusion the main reason for the lower LV volumes when comparing RT3DE and CMRI is the worse spatial resolution of RT3DE. The generation of RT3DE data-sets with best spatial and temporal resolution is also intensively discussed in human medicine [19, 30, 31]. When comparing both RT3DE programs there was no significant difference: however, the lowest LV volumes were attained when using 4D-TomTec™. These results are in agreement with a human study in awake patients with heart disease (17), whereas two other studies reported no significant differences between CMRI and different RT3DE analyzing programs [16, 18]. Manual contouring along the endocardial border should be more precise than semi-automatic tracing. However, contrary to the assumption that this manual optimization results in higher LV volumes even lower values were measured. This can be explained by the fact that the endocardial delineation was usually corrected further into the lumen of the ventricle, caused by the irregular ventricular surface. Consequently, a poor contrast behavior of the cardiac structures complicates endocardial definition. Perhaps contrast-enhanced 3D volumetric quantification could be a method for better border detection as shown in human medicine [32].

### LV function

Another part of the volumetric analysis is the evaluation of LV function by SV and EF. 4D-TomTec™ showed comparable LV function parameters with gold standard CMRI. This is due to systematic underestimation of EDV as well as ESV. Using 4D-AutLVQ™ there was no difference considering SV but the EF was significantly higher when measured by CMRI as opposed to 4D-TomTec™. Therefore, the evaluation of LV function by SV and EF by means of 4D-TomTec™ and 4D-AutLVQ™ is comparable with the gold standard CMRI.

### Measurement Time

A considerable disadvantage of manual volume correction is the significantly higher measurement time, which has been demonstrated in this study and is in agreement with human studies (15, 17). The measurement time with manual optimization using 4D-TomTec™ is significantly longer than using 4D-AutLVQ™ without any significant advantage regarding the measurement results. In contrast, using 4D-AutLVQ™ without manual correction is by far the quickest method, this technique underestimates the LV volumes even more than 4D-AutLVQ™ with manual correction. Consequently, with regard to measurement time and accuracy 4D-AutLVQ™ with manual optimization is the method with best agreement with CMRI.

### Reproducibility

The inter-observer variability 4D-AutLVQ™ (<11 %) is superior to 4D-TomTec™ (<20 %). This may be due to the more detailed options for LV ventricle depiction using 4D-TomTec™, resulting in higher variability of endocardial border detection caused by user dependent optimizations of contrast and brightness settings. Considering the different levels of experience (S.O.H. many years of experience, J.E. beginner) of the two observers the inter-observer variability itself is quite low. The excellent intra-observer variability (<3 %) using both measurement methods provides a further evidence that the inter-observer variability is caused by the differing assessment of the endocardial border by the two investigators and not by the software itself. Therefore, repeated measurements should be preferably performed by the same observer.

### Limitations

Limitations of this study are the low number of patients, as well as the exclusive examination of anesthetized healthy beagles. Also influences of prolonged anesthesia and of differences in patient positioning on measurement results cannot be ruled out. Furthermore reproducibility and repeatability was only determined for the analysis of the data and not for the acquisition of the data. Further studies examining awake dogs of different breeds and body-weights as well as of dogs with heart disease are necessary to evaluate the utility of RT3DE in veterinary cardiology.

## Conclusion

In summary, the results of this study demonstrate that both RT3DE volumetric analysis programs show underestimation of LV volumes with appropriate evaluation of LV function in comparison with gold standard CMRI. Considering duration of measurement and inter- and intra-observer variability time 4D-AutLVQ™ is superior to 4D-TomTec™. Therefore further studies of RT3DE in dogs should be performed preferably with 4D-AutLVQ™.

## Abbreviations

2/4Ch: Two-/ four-chamber
3DE: Three-dimensional echocardiography
CBC: Complete blood count
CHIEF: Canine Heart Failure International Expert Forum
CMRI: Cardiac magnetic resonance imaging
ECG: Electrocardiogram
EDV: End-diastolic volume
EF: Ejection fraction
ESV: End-systolic volume
Fig: Figure
LAX: Left ventricular outflow tract
LV: Left ventricle
RT3DE: Real-time three-dimensional echocardiography
SD: Standard deviation
Tab: Table

## Footnotes

1 Diazepam-ratiopharm® 10 mg/2 ml, Ratiopharm GmbH, Ulm, Germany.

2 L-Polamivet® 2.5/ 0.125 mg/ml; Intervet Deutschland GmbH, Unterschleißheim, Germany.

3 Narcofol® 10 mg/ml, CP- Pharma Handelsgesellschaft mbH, Burgdorf, Germany.

4 Tracheal Tube®, Smiths Medical. Kent, United Kingdom.

5 Isofluran CP®, CP- Pharma Handelsgesellschaft mbH, Burgdorf, Germany.

6 Dräger Ventilog 2, Dräger Medical AG, Lübeck, Germany.

7 GE Datex-Ohmeda, GE Healthcare, Helsinki, Finland.

8 Sterofundin®, Braun GmbH, Kronberg/Taunus, Germany.

9 Bair Hugger® Modell 505, Arizant Healthcare, Eden Prairie, MN, USA.

10 Vivid E9, GE Healthcare, Horten, Norway.

11 EchoPAC PC, 108.1.4 Version 110.x.x, GE Healthcare, Horten Norway.

12 4D LV-Function™ 2.2, TomTec Imaging Systems GmbH, Unterschleißheim.

13 4D-AutoLVQ™, EchoPAC PC, GE Healthcare, Horten, Norway.

14 Radio Translucent Foam Monitoring Electrodes, Philips, Eindhoven, The Netherlands.

15 SENSE Flex M 2 elements and SENSE Flex S 2 elements, Philips, Eindhoven, The Netherlands.

16 Achieva 3.0 T TX, Philips, Eindhoven, The Netherlands.

17 EWS R2.6.3.1., Philips, Eindhoven, The Netherlands.

18 Kardio-Analyse, Philips, Eindhoven, The Netherlands.

19 SAS version 9.3, SAS Institute, Cary, NC, USA.

20 GraphPad Prism, Version 5.00 for Windows, GraphPad Software, San Diego, Calif., USA.
